# Higher levels of anti-phosphorylcholine autoantibodies in early rheumatoid arthritis indicate lower risk of incident cardiovascular events

**DOI:** 10.1186/s13075-021-02581-0

**Published:** 2021-07-27

**Authors:** Sofia Ajeganova, Maria L. E. Andersson, Johan Frostegård, Ingiäld Hafström

**Affiliations:** 1 Division of Gastroenterology and Rheumatology, Department of Medicine Huddinge, Karolinska Institutet, 171 77 Stockholm, Sweden; 2 Department of Clinical Sciences, Rheumatology Division, Universitair Ziekenhuis Brussel, Vrije Universiteit Brussel, Brussels, Belgium; 3 Faculty of Medicine, Department of Rheumatology, Lund University, Lund and Spenshult Research and Development Centre, Halmstad, Sweden; 4 Section of Immunology and Chronic disease, Institute of Environmental Medicine, Karolinska Institutet, Stockholm, Sweden; 5 Rheumatology Unit, Karolinska University Hospital, Stockholm, Sweden

**Keywords:** Phosphorylcholine autoantibodies, Innate immunity, Cardiovascular events, Rheumatoid arthritis

## Abstract

**Background:**

The increased risk of cardiovascular events (CVE) in rheumatoid arthritis (RA) is not fully explained by traditional risk factors. Immuno-inflammatory mechanisms and autoantibodies could be involved in the pathogenesis of atherosclerotic disease. It has been suggested that anti-phosphorylcholine antibodies (anti-PC) of the IgM subclass may have atheroprotective effects. Here, we aimed to investigate the association between levels of IgM anti-PC antibodies with CVE in patients with early RA.

**Methods:**

The study population was derived from the BARFOT early RA cohort, recruited in 1994–1999. The outcome of incident CVE (AMI, angina pectoris, coronary intervention, ischemic stroke, TIA) was tracked through the Swedish Hospital Discharge and the National Cause of Death Registries. Sera collected at inclusion and the 2-year visit were analyzed with ELISA to determine levels of anti-PC IgM. The Kaplan-Meier estimates and Cox proportional hazards regression models were used to compare CV outcome in the groups categorized by baseline median level of IgM anti-PC.

**Results:**

In all, 653 patients with early RA, 68% women, mean (SD) age 54.8 (14.7) years, DAS28 5.2 (1.3), 68% seropositive, and without prevalent CVD, were included. During the follow-up of mean 11.7 years, 141 incident CVE were recorded.

Baseline IgM anti-PC above median was associated with a reduction in risk of incident CVE in patients aged below 55 years at inclusion, HR 0.360 (95% CI, 0.142–0.916); in males, HR 0.558 (0.325–0.958); in patients with BMI above 30 kg/m^2^, HR 0.235 (0.065–0.842); and in those who did not achieve DAS28 remission at 1 year, HR 0.592 (0.379–0.924). The pattern of associations was confirmed in the models with AUC IgM anti-PC over 2 years.

**Conclusion:**

Protective effects of higher levels of innate IgM anti-PC autoantibodies on CVE were detected in younger patients with RA and those at high risk of CVE: males, presence of obesity, and non-remission at 1 year.

## Introduction

The increased cardiovascular (CV) risk in rheumatoid arthritis (RA) is not fully explained by traditional risk factors and genetic markers [1, 2]. Inflammation plays a critical role in atherothrombosis [3]. Immuno-inflammatory mechanisms and autoantibodies could be involved in the pathogenesis of atherosclerotic disease [4, 5]. While most studies examine the risk factors of atherosclerosis and CV outcomes, studies focusing on the possible protective factors have been scarce.

Phosphorylcholine (PC), a component of phospholipids, is an important pro-inflammatory damage-associated compound, which is immunogenic if exposed as auto-antigens during stress, tissue damage, and inflammation [6, 7]. Anti-phosphorylcholine autoantibodies (anti-PC) have a role in maintaining the homeostasis of the immune system [8]. These autoantibodies serve “housekeeping” and could enhance clearance of damaged apoptotic cells, senescent IL-17+ T-cells, oxidized or otherwise modified lipoproteins, and induce intracellular blockade of inflammatory signaling cascades [7, 9].

Anti-PC of IgM subclass have atheroprotective effects and may play a role in the formation and stabilization of atherosclerotic plaque [8, 10]. Low levels of IgM anti-PC autoantibodies have been shown in association with increased risk of acute myocardial infarction and ischemic stroke and could predict CV disease (CVD) [11–13], which could be partly mediated by a fast carotid intima-media thickness (cIMT) progression [14]. Lower levels of IgM anti-PC autoantibodies have been reported in patients with RA who developed CVD and in SLE patients with carotid atherosclerotic plaques [15, 16]. In contrast, high levels of IgM anti-PC may be protective and associate with a reduced rate of atherosclerosis progression [17, 18].

Whether the effects of innate immunity are dependent on the individual risk characteristics is unknown. If protective effects of anti-PC autoantibodies differ in the patient settings, this might explain that some studies observed an association between levels of anti-PC and risk of CV events (CVE) [19], while others did not [20]. Indeed, an unfavorable effect of low anti-PC on cIMT progression and risk of CVE has been evident in men, but not in women [14]. Further, the relation between high levels of IgM anti-PC and a decreased rate of cIMT progression has been observed among patients with hypertension [21]. Association between IgM anti-PC autoantibodies and pro-atherogenic T cell subsets in SLE has been shown in patients with high triglyceride or low HDL levels, but not in those with a normal lipid profile [22].

We hence hypothesized that the association between innate immunity and atherosclerosis and, therefore, benefits of CV risk reduction in relation to high levels of IgM anti-PC could be different depending on the CV risk level of the studied population. The aim of the present analysis was to investigate the association between IgM anti-PC autoantibodies with CV morbidity in a large cohort of patients with early RA within the groups defined by demographics and CV risk characteristics.

## Patients and methods

### Patients and outcome assessment

The study population was derived from the prospective observational BARFOT (Better Anti-Rheumatic PharmacO Therapy) cohort from secondary care in southern Sweden with newly diagnosed early (symptom duration ≤ 1 year) RA according to the ACR 1987 criteria [23]. Patients were enrolled between 1993 and 1999. Treatment was started and adjusted during follow-up by the treating rheumatologist in accordance with the current recommendations. For a further cohort description, see elsewhere [24].

The study participants provided written informed consent. The study was approved by the local ethics committees, EPN Stockholm 2008/840-31 and 2011/381-31/4, and was performed in accordance with the Declaration of Helsinki.

### Data collection

Of 780 eligible patients aged > 18 years without prevalent CV event prior to inclusion, 653 patients with available blood samples at baseline for analysis of anti-PC autoantibodies were included in this study. Of these, 523 had anti-PC antibodies measured both at baseline and follow-up at 2 years. Baseline characteristics of the patients with available blood samples and those without were similar as to age, sex, main disease characteristics, and CV risk factors.

Demographics, body mass index (BMI), laboratory data, and information on DMARDs were obtained from the BARFOT database. The following CV risk factors were registered at inclusion: smoking history, self-reported history or medication prescription for hypertension, diabetes mellitus, and hyperlipidemia.

RA disease activity was calculated using the Disease Activity Score for 28 joints (DAS28) with ESR [25]. Disease remission was defined as DAS28 < 2.6 [26]. Functional status was self-assessed by the validated Swedish version of the Stanford Health Assessment Questionnaire (HAQ) [27].

Antibodies to cyclic citrullinated peptides (anti-CCP) were detected using the enzyme-linked immunosorbent assay (ELISA) CCP2 test (Euro-Diagnostica, Malmö, Sweden), and a level > 25 IU/ml was regarded as positive. IgM RF was measured by agglutination test (Serodia, Fujirebio, Tokyo, Japan), and a level > 20 units/ml was defined as positive. Seropositivity was defined by the presence of RF and/or anti-CCP. IgM anti-PC autoantibodies were determined in serum samples collected at baseline and at 2 years using ELISA (Athera CVDefine kit, Athera Biotechnologies AB, Stockholm, Sweden) in accordance with the manufacturer’s instructions [12].

### Outcome assessment

The outcome was CVE such as fatal or non-fatal myocardial infarction, angina pectoris, coronary intervention, ischemic stroke, and transient ischemic attack (TIA). CV morbidity data were identified through the Swedish Hospital Discharge Registry from 1987 and the National Cause of Death Registries through 2010. Patients contributed at-risk time from the date of entry into the cohort. Censoring date occurred at the date of first-ever incident CV event, or death, or censoring date of December 2010, whichever occurred first.

### Statistical methods

Descriptive statistics are reported as means (SD) or medians (IQR), as suitable, for continuous variables, and percentages for categorical variables. To compare medians between the groups, a Mann-Whitney *U* test for independent samples was used. The area under the curve (AUC) was calculated for the measures of IgM anti-PC assessed at inclusion and after 2 years.

The incidence CV rates with the 95% confidence interval (CI) for a Poisson count were presented as events per 100 person-years at risk. The clinically relevant cutoffs of novel IgM anti-PC autoantibodies are not established. Before performing analysis, based on previous results and in order to have reasonably large groups for survival analysis, we have chosen an arbitrary threshold at median IgM anti-PC levels for this study. Rates of event-free survival in patients in total and per group divided by median IgM anti-PC (higher IgM anti-PC levels vs. lower levels) were compared using a Kaplan-Meier analysis. Equality of time-to-event function between the groups was tested with a log-rank test. Relative hazard ratios from Cox regression models were used to estimate the effect of IgM anti-PC on the outcome in total patient population and within the groups categorized by sex, ages (above the median age of 55 years at inclusion vs. below the median age of 55 years), traditional risk factors, and disease characteristics with progressive adjustment if a *p* < 0.10: unadjusted, adjusted for age and sex, and further fully adjusted for traditional CV risk factors (BMI, smoking, hypertension, diabetes mellitus, hyperlipidemia).

Significance tests were two-tailed and conducted at the 0.05 level of significance. IBM SPSS, version 26 (SPSS Inc., Chicago, IL), was used for the analyses.

## Results

Baseline characteristics of 653 included patients with early RA are summarized in Table 1. Their mean age was 55 years, 68% were women, 68% were seropositive, and the mean DAS28 was 5.2. Of all, 29% of the patients were current smokers, 15% had hypertension, and only 4% and 1% of the patients reported history or treatment for diabetes and hyperlipidemia, respectively. A good half of the patients received MTX and glucocorticoids, and only 2.4% of the patients could receive biologic DMARDs in the first 2 years. DAS28 remission was achieved in 36% of the cohort at 1 year and in 40% at the 2-year follow-up.

**Table 1:** Characteristics in 653 patients with rheumatoid arthritis

Characteristics at baseline	
Age, years	54.8 (14.7)
Women, %	68
BMI, kg/m^2^	25.5 (5.1)
Smoking current, %	29
Hypertension, %	15
Diabetes mellitus, %	4
Hyperlipidemia, %	1
Symptom duration, months	6.3 (3.2)
Seropositive, %	68
DAS28	5.16 (1.27)
HAQ	0.99 (0.63)
IgM Anti-PC, U/ml	60.9 (36.4–94.9)
At 1 year	
DAS28 remission, %	36.3
Use of MTX the first year, %	50
Use of glucocorticosteroids the first year, %	51
At 2 years	
DAS28 remission, %	39.5
Use of MTX first 2 years, %	58
Use of glucocorticosteroids first 2 years, %	53
Use of biological first 2 years, %	2.4
IgM Anti-PC, U/ml	56.0 (32.4–84.2)
IgM Anti-PC AUC per 2 years, U/ml	117.3 (68.8–201.4)

Values are reported as mean (SD), median (IQR), or percentage

*BMI*, body mass index; *Seropositive*, RF and/or ACPA positive; *DAS28*, Disease Activity Score 28-joint count; *DAS28 remission*, if DAS28 < 2.6; *HAQ*, Health Assessment Questionnaire; *Anti-PC*, phosphorylcholine autoantibodies; *MTX*, methotrexate

Median (IQR) IgM anti-PC decreased from baseline levels of 60.9 (36.4–94.9) U/ml to 56.0 (32.3–84.2) at 2 years, *p* < 0.001. The baseline and 2-year follow-up levels of IgM anti-PC were lower in patients with CV outcome than in those without, *p* = 0.020 and *p* = 0.012, respectively. Baseline median IgM anti-PC levels were also statistically significantly lower in patients older than 55 years old at inclusion, in males, in patients with BMI above 30 kg/m^2^, and in those with hypertension (Table 2).

**Table 2:** IgM anti-PC autoantibody baseline distribution (median) according to characteristics of patients with rheumatoid arthritis

		IgM anti-PC, U/ml, baseline distribution		
		*n*	Median	*p* value
CV events during follow-up	Non-CV	512	64.19	0.020
	CV	141	51.79	
Age, years	< 55	317	69.21	< 0.001
	> 55	336	53.20	
Sex	Female	443	64.81	< 0.001
	Male	210	52.90	
BMI, kg/m^2^	< 25	334	67.28	0.002
	25–30	235	55.02	
	> 30	84	47.56	
Smoking	Non-smoker	463	58.34	0.070
	Current smoker	190	65.0	
Hypertension	Non-hypertensive	554	62.68	0.013
	Hypertensive	99	47.91	
Diabetes mellitus	Non-diabetic	627	60.89	0.631
	Diabetic	26	56.98	
Hyperlipidemia	Non-hyperlipidemic	648	60.88	0.658
	Hyperlipidemic	5	48.17	
Seropositive	Seropositive	441	63.59	0.190
	Seronegative	212	55.35	
DAS28 remission at 1 year	Remission	237	61.11	0.895
	Non-remission	416	60.10	
DAS28 remission at 2 years	Remission	256	61.73	0.223
	Non-remission	397	57.97	

Statistical significance was tested with the Mann-Whitney *U* test

*BMI*, body mass index; *DAS28*, Disease Activity Score 28-joint count; *DAS28 remission*, if DAS28 < 2.6; *HAQ*, Health Assessment Questionnaire; *Anti-PC*, phosphorylcholine antibodies

During an 11.7-year mean follow-up, the patients experienced 141 first CVE (21.6%), corresponding to an incidence rate of 1.85 events per 100 person-years (95% CI, 1.54–2.15). Of these, ischemic coronary events were censored in 73 cases, ischemic cerebrovascular events in 49, and both coronary and cerebrovascular events in 19 cases.

### Outcome of incident CV events in relation to IgM anti-PC

**Fig. 1:**
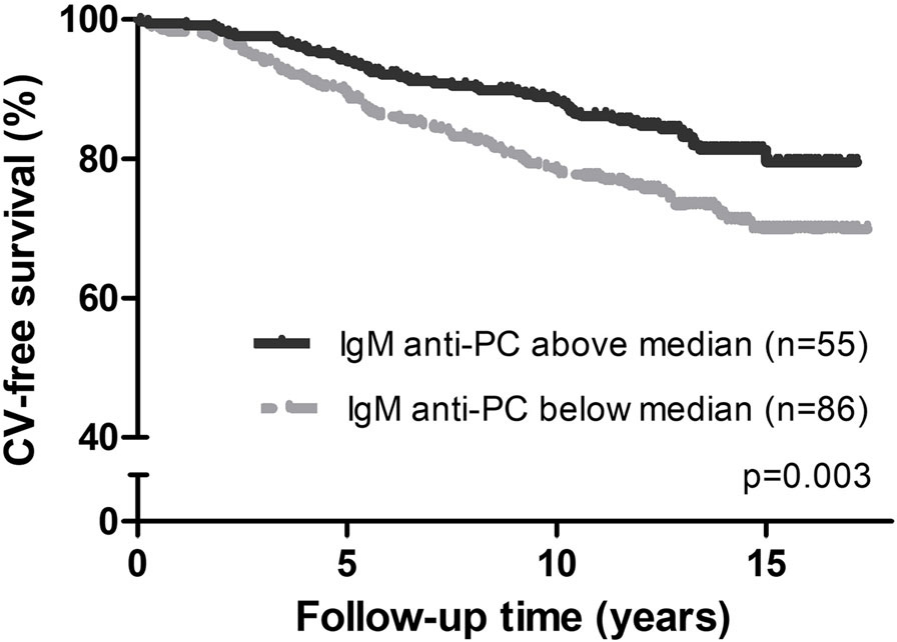
Kaplan-Meier estimates of CV-free survival in rheumatoid arthritis according to baseline IgM anti-PC levels. Kaplan-Meier curves in the total cohort, *p* values by log-rank test, and number (*n*) of CV outcomes recorded in the groups categorized by baseline IgM anti-PC levels above and below median

First, the association between autoantibodies IgM anti-PC and CV outcome was studied. This analysis showed that event-free times were significantly better among patients with the baseline levels of IgM anti-PC above median compared with those below median, *p* = 0.003 by the log-rank test (Fig. 1). The higher levels of IgM anti-PC were associated with a 40% reduction in CV risk, HR 0.603 (95% CI, 0.430–0.846) (Table 3).

**Table 3:** Association between IgM anti-PC and incident CVE, in total and within groups by risk characteristics

	*N* of events	Non-adjusted HR (95% CI)	*p*	Adjusted HR (95% CI)	*p*
**IgM anti-PC baseline** **> median vs. < median**	141	0.603 (0.430–0.846)	0.003	0.828 (0.589–1.165)	0.279
Analyses within strata					
Age					
Age < 55 years	22	0.396 (0.166–0.943)	0.036	0.360 (0.142–0.916)†	0.032
Age > 55 years	119	0.883 (0.611–1.277)	0.510	–	
Sex					
Women	81	0.608 (0.391–0.945)	0.027	0.985 (0.629–1.541)	0.946
Men	60	0.614 (0.359–1.050)	0.075	0.558 (0.325–0.958)†	0.034
BMI					
BMI < 25 kg/m^2^	63	0.524 (0.316–0.868)	0.012	0.827 (0.496–1.377)	0.465
BMI 25–30 kg/m^2^	59	0.885 (0.523–1.498)	0.650	–	
BMI > 30 kg/m^2^	19	0.278 (0.080–0.969)	0.044	0.235 (0.065–0.842)†	0.026
Smoking					
Non-smoker	98	0.510 (0.337–0.774)	0.002	0.722 (0.475–1.098)	0.128
Current smoker	43	0.877 (0.482–1.597)	0.668	–	
Hypertension					
Non-hypertensive	104	0.594 (0.402–0.880)	0.009	0.878 (0.591–1.305)	0.520
Hypertensive	37	0.805 (0.410–1.583)	0.530	–	
Diabetes mellitus					
Non-diabetic	133	0.663 (0.469–0.936)	0.019	0.899 (0.635–1.273)	0.549
Diabetic	8	0.017 (0.001–5.059)	0.161	–	
Hyperlipidemia					
Non-hyperlipidemic	3	0.601 (0.427–0.847)	0.004	0.836 (0.592–1.179)	0.307
Hyperlipidemic	138	1.423 (0.126–16.043)	0.775	–	
Seropositivity					
Seropositive	94	0.669 (0.444–1.007)	0.054	0.846 (0.561–1.276)	0.425
Seronegative	47	0.496 (0.265–0.927)	0.028	0.782 (0.415–1.473)	0.782
DAS28 remission at 1 year					
Remission	49	1.235 (0.691–2.206)	0.476	–	
Non-remission	92	0.440 (0.284–0.683)	< 0.001	0.592 (0.379–0.924)†	0.021
DAS28 remission at 2 years					
Remission	54	0.851 (0.498–1.457)	0.557	–	
Non-remission	87	0.498 (0.320–0.774)	0.002	0.681 (0.435–1.068)†	0.095
**AUC of IgM anti-PC at baseline and 2 years** **> median vs. < median**	116	0.614 (0.423–0.890)	0.010	0.847 (0.583–1.232)	0.386
Age					
Age < 55 years	16	0.520 (0.194–1.397)	0.195	–	
Age > 55 years	100	0.841 (0.562–1.257)	0.398	–	
Sex					
Women	67	0.765 (0.474–1.236)	0.274	–	
Men	49	0.490 (0.264–0.911)	0.024	0.428 (0.227–0.807)†	0.009
BMI					
BMI < 25 kg/m^2^	50	0.493 (0.279–0.871)	0.015	0.841 (0.471–1.503)	0.559
BMI 25–30 kg/m^2^	48	0.973 (0.547–1.730)	0.926	–	
BMI > 30 kg/m^2^	18	0.330 (0.105–1.031)	0.056	0.297 (0.089–0.988)†	0.048
DAS28 remission at 1 year					
Remission	40	1.121 (0.591–2,124)	0.727	–	
Non-remission	76	0.487 (0.300–0.791)	0.004	0.648 (0.395–1.064)†	0.087
DAS28 remission at 2 years					
Remission	44	0.856 (0.473–1.549)	0.607	–	
Non-remission	72	0.510 (0.314–0.828)	0.006	0.698 (0.430–1.136)	0.148

Values are hazard ratios (HRs) with 95% CI obtained with Cox proportional hazard regression. The number of patients with measures of IgM anti-PC at baseline was 653, and the number of patients with measures of AUC of IgM anti-PC was 523. Anti-PC baseline median ≥ 60.9 U/ml, AUC of anti-PC median ≥ 117.3 U/ml

*CVE*, cardiovascular events; *BMI*, body mass index; *DAS28*, Disease Activity Score 28-joint count; *DAS28 remission*, if DAS28 < 2.6; *HAQ*, Health Assessment Questionnaire; *Anti-PC*, phosphorylcholine antibodies

Adjusted for age and sex. †HR, a fully adjusted (for age, sex, BMI, smoking, hypertension, diabetes mellitus, hyperlipidemia) analysis was run if results of the analyses adjusted for age and sex showed a *p* < 0.10

**Fig. 2:**
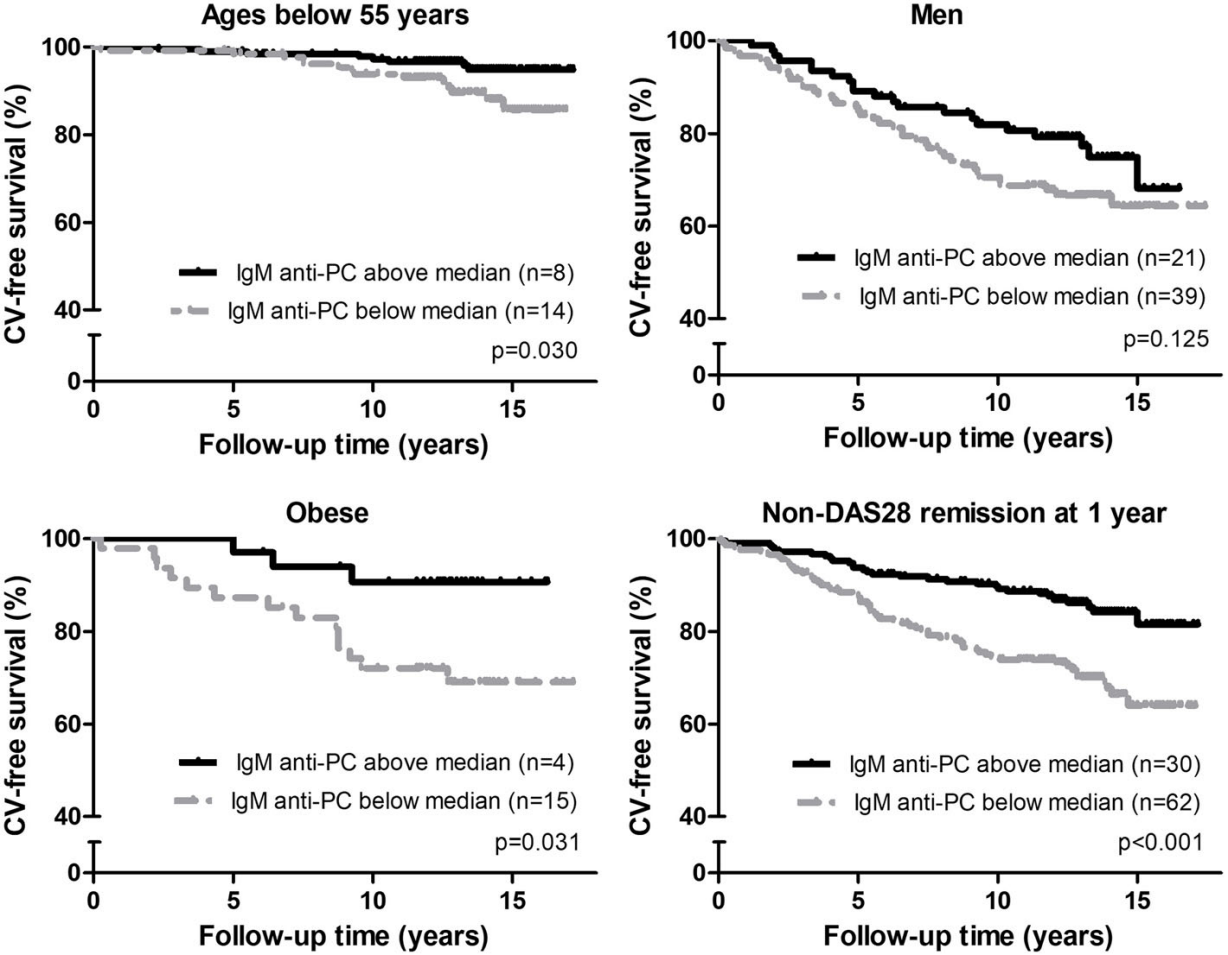
Kaplan-Meier estimates of CV-free survival within the patient groups as indicated. Kaplan-Meier curves within the patients with rheumatoid arthritis aged below 55 years at inclusion, men, obese, and in those who did not achieve DAS28 remission at 1-year follow-up, *p* values by log-rank test, and number (*n*) of CV outcomes recorded in the groups categorized by baseline IgM anti-PC levels above and below the median

We hypothesized that the association between IgM anti-PC and the risk of CV outcome is different in the presence of CV risk factors. We therefore further performed analyses within the groups categorized by risk factors (Table 3). In unadjusted survival analyses, the risk of CV was lower in patients with the baseline levels of IgM anti-PC above median in those aged below 55 years, of both sexes, BMI below 25 kg/m^2^ and above 30 kg/m^2^, without traditional risk factors (non-smokers, non-hypertensives, non-hyperlipidemic), seronegative, and among those who did not achieve DAS28 remission at 1-year and 2-year follow-up. In multivariate analyses, baseline IgM anti-PC above median was significantly associated with decreased risk of CV outcome in patients aged below 55 years at inclusion, HR 0.360 (95% CI, 0.142–0.916); in males, HR 0.558 (95% CI, 0.325–0.958); in those with BMI above 30 kg/m^2^, HR 0.235 (95% CI, 0.065–0.842); and among those who did not achieve DAS28 remission at 1 year, HR 0.592 (95% CI, 0.379–0.924) (Table 3, Fig. 2).

Similarly, applying the integrated measurement of IgM anti-PC over 2 years, the higher levels above median of AUC IgM anti-PC were associated with reduced CV risk, HR 0.614 (95% CI, 0.423–0.890). The levels above median of AUC IgM anti-PC were independently associated with a CV risk reduction in males, HR 0.428 (95% CI, 0.227–0.807), and in patients with BMI above 30 kg/m^2^, HR 0.297 (95% CI, 0.089–0.988). Likely because of lower numbers in analysis and a shorter observation period, the association between the higher AUC IgM anti-PC and a better CV outcome was attenuated in patients not in DAS28 remission, adjusted HR 0.648 (95% CI, 0.395–1.064) (Table 3). This pattern of associations in males, in patients with a higher BMI, and among those not in DAS28 remission the first 2 years was confirmed in the models with IgM anti-PC measurement at 2-year follow-up (data not shown).

## Discussion

In this study in patients with early RA, we observed that higher levels of IgM anti-PC autoantibodies, compared with lower levels, were associated with fewer incident CVE over more than 10 years. The benefits of higher IgM anti-PC levels for the reduction of CV risk were shown in younger patients, in males, and in the presence of obesity and if DAS28 remission was not achieved after 1 year. These findings suggest that the protective function of innate immunity could be primarily detected in patients at high risk of CVE. This points out to an alternative pathway in atherothrombosis which may be activated in the presence of additional pro-inflammatory factors, thus intersecting with traditional pathways.

A large body of evidence supports a role for chronic inflammation in atherogenesis, and autoantibodies have been identified as mediators in the complex inflammatory environment. The concept of autoimmune diseases suggests that autoantibodies are inherently pathogenic; however, some autoantibodies of innate “natural” immunity are protective, i.e., “protective autoimmunity.” Innate IgM autoantibodies could arise spontaneously without antigenic or microbial stimuli. These IgM autoantibodies are present in the circulation from birth [28], and they are also developed by exposure to the phospholipids, such as phosphorylcholine (PC) [29]. PC is present in apoptotic cells, and they act as enhancers of the inflammation underlying both atherosclerosis and autoimmunity [30, 31]. IgM anti-PC antibodies recognizing and increasing the clearance of dying cells have been postulated as regulators of the inflammatory pathways possibly driving immunosenescence and atherogenesis [22, 32].

We here confirmed and extended the previous findings of lower levels of IgM anti-PC autoantibodies in men compared to women, and higher levels of these autoantibodies in younger patients compared to older ones [13, 14, 21]. An interesting question is whether atherosclerosis should be seen as a normal part of human aging or as a pathological process which could be, if not abolished, at least strongly reduced. The underlying mechanisms that cause decreased IgM anti-PC levels could have different explanations including both lifestyle factors, differences in diet, exposure to infections, and underlying genetic variations. However, both among New Guineans with a traditional lifestyle and in Swedish controls with a western lifestyle, men had significantly lower levels of IgM anti-PC as compared to women [33]. It thus appears that low levels of IgM anti-PC are likely a part of predisposition for atherosclerosis in males. Genetic factors could also play an additional role since the heritability of anti-PC is 37% [34]. Given the biological role of IgM anti-PC in modulation and anti-atherogenic mechanisms, it is tempting to hypothesize that high levels of anti-PC may slow down the progression of atherosclerosis and may facilitate the stabilization of plaque. Indeed, the lower levels of IgM anti-PC have been demonstrated in patients with SLE in the presence of carotid atherosclerotic plaques [16] and also in individuals with the fastest progression of cIMT and carotid plaque measurements [14].

In addition, our data showed an association between higher levels of IgM anti-PC autoantibodies and risk reduction of CVE in men and younger patients with early RA. The findings here consistent with previous studies showing a sex difference in the risk of CVE associated with IgM anti-PC antibodies [13, 14]. Both in the current analysis and an earlier large epidemiological study [13], the association between IgM anti-PC and risk of CVE was not affected by the presence of traditional risk factors, suggesting that activation of innate immunity pathway might be independent of traditional pathways. Meanwhile, IgM anti-PC autoantibodies may be of particular interest for risk stratification in some patient groups, such as at younger ages. The reduction of CV risk in younger patients in association with higher levels of IgM anti-PC autoantibodies reported herein might reflect the age-related variation in plaque composition and plaque remodeling mechanisms at different ages [35]. In line with here reported findings, both the faster progression of carotid atherosclerosis and the higher risk of CVE in association with low anti-PC levels have been shown in younger individuals, whereas this association was not found in the older ones [14]. It thus seems that low levels of IgM anti-PC could be a part of human senescence. Also, in the study on individuals from New Guinea, IgM anti-PC was lower in New Guineans over the age of 40 compared to those under 40 years old and decreased with age among Swedish controls above 40 years old [33]. Although the results from these studies cannot provide definitive answers for a sex and age difference in the risk of CVE associated with IgM anti-PC antibodies, it is possible that innate immunity state could be one of the underlying factors of the known lower risk of CVD in women as compared to men of similar age and in younger individuals compared to older ones.

The underlying mechanism by which the protective innate immunity pathway could be activated is not clear. One of the explanations could be a direct effect on the endothelium, which could become dysfunctional as a response to injury, such as oxidative stress, leading to pro-inflammatory immunogenic effects. IgM anti-PC and other phospholipids could have dual immunomodulatory effects: they might be protective by neutralizing the inflammatory effects by initiation and progression of atherosclerosis, as well as ensuing plaque rupture, while low levels of these autoantibodies could be per se a cause of inflammation in the context of oxidative stress [4]. The protective effect of some gene polymorphisms on the risk of atherosclerotic disease has been reported in anti-CCP-negative patients with RA [36, 37]. Interestingly, in unadjusted analysis herein, the higher levels of IgM anti-PC were associated with a reduction of CV risk in the group of anti-CCP-negative patients. Whether IgM anti-PC antibodies may be a part of the explanation of lower CV risk in seronegative RA and whether genetic determinants and epigenetic modifications are involved in the activation of the atheroprotective function of these antibodies are not known.

In this analysis, levels of IgM anti-PC antibodies were lower in patients with BMI above 30 kg/m^2^, which is consistent with previous reports in SLE patients [38]. Obesity is associated with an increased risk of metabolic syndrome and CVE within most westernized populations, but it also seems that fat distribution, lean mass, and cardio fitness, i.e., adiposity and cachexia, could play an essential role in determining morbidity. Abundant data indicate the presence of increased pro-inflammatory adipose tissue macrophages, as well as other immune cells, and decreased number, or proportion, of adipose tissue Treg cells, in obesity [39], which, in turn, could cause inflammation and contributes critically to atherothrombosis. Innate immunity may play an additional role in modulating metabolic and pro-inflammatory consequences in obesity, i.e., immunometabolism. Even though it remains to be shown, it is possible that direct and indirect effects of adiposity on inflammatory cells could induce a hypoxic state promoting inflammation and endothelial dysfunction. In the clinical setting, the levels of anti-PC IgM were lower in patients with rheumatoid cachexia than in those without [40]. Interestingly, no correlation has been observed between levels of IgM anti-PC, LDL cholesterol, HDL cholesterol, and hsCRP [14], in line with an earlier report of an independent association between low IgM anti-PC and bilateral carotid plaque in patients with RA [41]. Altogether, it suggests an alternative pathway of atheroprotective effects of IgM anti-PC, independent of the traditional hyperlipidemic pathway.

The data presented herein showed a reduction of CV risk in association with high levels of IgM anti-PC in patients with BMI above 30 kg/m^2^, suggesting high levels of innate autoantibodies as a protective factor in obesity. An important provisional hypothesis deriving from the current analysis is that high levels of IgM anti-PC autoantibodies could “compete out” CVE risk, at least to some extent, in obese patients through the protective innate immunity pathway. Importantly, diet and physical activity may influence IgM anti-PC in RA. The dietary studies have reported the very high levels of anti-PC in patients with RA on a Mediterranean diet, increased levels of anti-PC when changing from a Western diet to a gluten-free vegan diet [40, 42], and also low levels of IgM anti-PC in association with low physical activity [43]. Given the atheroprotective effects of higher levels of IgM anti-PC, lifestyle and physical activity recommendations should be encouraged.

Finally, the intriguing finding of this study is that higher levels of IgM anti-PC autoantibodies were protective in patients who were not in DAS28 remission at 1-year follow-up. This observation is likely consistent with the oxidative stress hypothesis of activation of protective innate immunity pathway, a means for the host to survive in a setting of chronic inflammation [30]. It has long been stated that both RA and atherosclerosis share similar underlying inflammatory pathways [3]. Disease activity is one of the contributing risk factors for increased atherosclerosis and CVD in patients with RA. Tight-control treatment and additional benefits of sustained disease remission, as compared to low disease activity, are associated with normalized survival in patients with RA [44].

Although herein we could not detect differences in the distribution of IgM anti-PC autoantibodies by disease activity among patients with early RA, in the previous cross-sectional study of established RA, patients with DAS28 remission after 12 months, compared to those not in remission, had higher baseline anti-PC levels [45]. These findings complement previous work in which low levels of IgM anti-PC autoantibodies have been demonstrated in patients with RA who experienced a subsequent CVE [15]. The studies in SLE have shown a correlation between increased levels of IgM and a lower disease activity along with damage scores, the reduced presence of carotid artery plaques, and reduced frequency of CVE [16, 46]. Low IgM anti-PC levels may characterize an immunodeficiency and chronic inflammation not captured by conventional laboratory methods [45]. Indeed, a previous study has found that IgM anti-PC autoantibodies significantly increased during 1-year anti-TNF treatment in patients with RA, possibly through a direct inhibitory effect of TNF on B-cells producing anti-PC, or indirectly through decreased systemic inflammation [45]. Immunomodulatory drugs, such as methotrexate and anti-TNF treatments, have been associated with improved endothelial function and CV outcome in RA [47, 48]. In this context, an interesting therapeutic question is whether amelioration of autoimmunity could also ameliorate atherosclerosis. Immunization with a vaccine containing PC could have provided proof of principle that an alternative innate immunity pathway can prevent atherosclerotic events.

Although we cannot provide direct evidence of drug effects in this study, DAS28 remission could likely give an estimation of conventional treatment effects in the patient cohort sampled before a broad use of biologicals. Because of the small numbers of patients in the subgroups, relevant effects of IgM anti-PC in the presence of some risk factors may not have been detected. At the same time, the strength of the study is its sample size and prospective design with long-term follow-up sufficient to study CV outcomes.

## Conclusion

Our data suggest the benefits of higher levels of innate IgM anti-PC autoantibodies in the reduction of CV risk in early RA, in particular in younger patients and in those at high risk of CVE. This study supports the novel hypothesis of “protective autoimmunity” and emphasizes the potential value of an alternative innate anti-inflammatory mechanism in atheroprotection and chronic inflammation, which might be activated in the presence of additional pro-inflammatory factors. We believe that closer investigation into the underlying mechanisms by which innate immunity protects against the risk of CVE represents an important evolving area that may have diagnostic and therapeutic implications beyond rheumatology.

## Data Availability

All data relevant to the study are included in the article.

## Abbreviations

ACR: American College of Rheumatology
Anti-CCP: Antibodies to cyclic citrullinated peptides
Anti-PC: Anti-phosphorylcholine autoantibodies
AUC: Area under the curve
BARFOT: Better Anti-Rheumatic PharmacO Therapy
BMI: Body mass index
CI: Confidence interval
cIMT: Carotid intima-media thickness
CRP: C-reactive protein
CV: Cardiovascular
CVD: Cardiovascular disease
CVE: Cardiovascular events
DAS28: Disease Activity Score for 28 joints
DMARD: Disease-modifying anti-rheumatic drug
ELISA: Enzyme-linked immunosorbent assay
ESR: Erythrocyte sedimentation rate
HAQ: Health Assessment Questionnaire
HDL: High-density lipoprotein
HR: Hazard ratio
IQR: Interquartile range
LDL: Low-density lipoprotein
MTX: Methotrexate
PC: Phosphorylcholine
RA: Rheumatoid arthritis
RF: Rheumatoid factor
SD: Standard deviation
SLE: Systemic lupus erythematosus
TIA: Transient ischemic attack
